# Pregnancy outcomes of 4,200 fetuses with increased nuchal translucency in Henan, China

**DOI:** 10.3389/fmed.2025.1514504

**Published:** 2025-04-02

**Authors:** Zhenglong Guo, Wenke Yang, Qiongrui Zhao, Yuwei Zhang, Xin Wang, Jinming Wang, Ruili Wang, Bingtao Hao, Shixiu Liao

**Affiliations:** ^1^Henan Provincial Key Laboratory of Genetic Diseases and Functional Genomics & National Health Commission Key Laboratory of Birth Defects Prevention, Medical Genetics Institute of Henan Province, Henan Provincial People’s Hospital, People’s Hospital of Zhengzhou University, Zhengzhou, China; ^2^Department of Ultrasound, Henan Provincial People’s Hospital, Zhengzhou, China

**Keywords:** nuchal translucency thickness, pregnancy outcome, first-trimester screening, chromosome abnormalities, ultrasound soft indicators

## Abstract

**Background and objective:**

Increased nuchal translucency (NT) thickness measured at 11–14 weeks of gestation in fetuses has been linked to adverse pregnancy outcomes. This study aimed to evaluate pregnancy outcomes in fetuses with NT ≥3.0 mm.

**Methods:**

This retrospective analysis included 4,200 singleton pregnancies diagnosed with increased NT thickness (≥3.0 mm) through first-trimester ultrasound screening across 76 hospitals in Henan Province from 2017 to 2021. Follow-up on pregnancy outcomes was completed through telephone interviews and electronic medical records.

**Results:**

Among the 4,200 pregnancies with NT ≥3.0 mm, adverse pregnancy outcomes were observed in 31.5% of the fetuses. These outcomes included elective termination of pregnancy (TOP), spontaneous abortion (SA), threatened abortion (TA), and live birth with malformations. A total of 547 fetuses underwent further examination through karyotype analysis after genetic counseling, revealing that 10.2% were aneuploid, primarily due to Trisomy 21 (7.1%).

**Conclusion:**

The incidence of increased NT in our study was 0.49%, which was associated with chromosomal abnormalities and developmental disorders, leading to an increased risk of adverse pregnancy outcomes. Abnormal ultrasound soft markers, along with NT > 4 mm, may further elevate the risk of adverse pregnancy outcomes. These findings should be taken seriously in the context of further prenatal diagnosis for fetuses with increased NT.

## Introduction

Fetal nuchal translucency (NT), as visualized by ultrasonography, refers to a temporary subcutaneous accumulation of fluid located behind the fetal neck and back, typically resolved by 14 weeks of gestation (1, 2). Numerous studies indicate that an increase in NT thickness is frequently associated with chromosomal abnormalities (3, 4), major cardiac defects (5, 6), neurodevelopmental disorders (7), skeletal dysplasia (8), spinal muscular atrophy (9), and various other genetic disorders and syndromes. These chromosomal abnormalities and structural malformations are significantly correlated with adverse pregnancy outcomes (10–13).

The first-trimester NT ultrasound examination, either alone or in conjunction with maternal serum biomarkers, serves as an effective method for early screening of fetal malformations, particularly trisomy 21 (14–17). Despite China’s large population and high incidence of birth defects (18, 19), limited research exists regarding the correlation between increased NT and pregnancy outcomes among pregnant women. Consequently, we conducted a retrospective cohort study to investigate this correlation. The study was carried out across multiple centers in Henan Province, the third largest province in China.

## Methods

### Data source

Since 2017, Henan Province has implemented a free prenatal screening initiative aimed at reducing the incidence of birth defects and enhancing the quality of the birth population. A key component of this initiative is the provision of free first-trimester ultrasound screening, commonly referred to as nuchal translucency (NT) screening, for pregnant women throughout the province. To ensure high-quality assessments, ultrasound physicians are required to undergo specialized technical training and obtain a maternal and infant healthcare technical assessment certificate. The screening is conducted on fetuses with a crown-rump length (CRL) ranging from 45 to 84 mm, corresponding to a gestational age of 11 to 13 weeks and 6 days. Transabdominal scans are conducted using either a 1–6 MHz or a 2–9 MHz probe. Participation in the screening necessitates that pregnant women provide signed informed consent. The primary examination includes evaluating the number of fetuses, fetal heartbeat, NT thickness, CRL, placental position, and amniotic fluid volume. In measuring NT thickness, three measurements are taken in the midsagittal section of the fetal head, neck, and upper chest, with the maximum value recorded.

### Follow-up

Pregnant women with an NT ≥ 3 mm, suspected of developmental abnormalities during initial screening, should undergo further evaluation. A referral form must be issued to facilitate the transfer of these high-risk pregnant women to the appropriate institution for additional prenatal diagnosis through amniocentesis, in accordance with the written recommendations provided by the screening agency. Furthermore, all pregnant women with increased NT should be monitored throughout the postpartum period as necessary. Fetal outcome data for these cases were collected through questionnaire interviews, using the same methodologies as those previously published (20). This process included three follow-up visits at 20–24 weeks of gestation, 28–34 weeks of gestation, and 6 months postpartum. The Henan Provincial People’s Hospital serves as a prenatal diagnosis institution and is responsible for quality management and prenatal diagnosis within the free prenatal screening projects conducted by 76 prenatal screening centers in the southeastern region of Henan Province.

### Data analysis

Data analysis and graphing were performed using SPSS version 25.0 and MedCalc version 15.2.2. Continuous variables are expressed as means ± standard deviations (SDs) and were compared using *t*-tests. Categorical data are presented as case counts, with the chi-square test performing percentage comparisons. Fisher’s exact probability method was applied when expected values were less than one. Receiver operating characteristic (ROC) curve analysis was used to assess the diagnostic value of indicators such as nuchal translucency (NT) thickness and maternal age, which facilitated the determination of optimal thresholds, as well as sensitivity and specificity. A significance level of *p* < 0.05 was considered statistically significant.

## Results

### Pregnancies with NT ≥ 3 mm

This study included 100,910 singleton pregnancies screened for NT between 1 July 2017 and 31 December 2021. Among these, 4,899 fetuses were identified with increased NT thickness (≥3.0 mm) through the electronic medical records system, resulting in an overall incidence of 0.49% over the 5-year period (see Table 1). We analyzed the pregnancy outcomes of these fetuses by synthesizing the results from three follow-up visits documented in the system. In 2022, a comprehensive telephone review was conducted to validate the follow-up results. Unfortunately, we lost contact with or received refusals from family members in 699 cases. Table 2 presents basic information for the remaining 4,200 fetuses and pregnant women. The mean (±SD) crown-rump length (CRL) and gestational age were 59.15 ± 8.56 mm and 12.33 ± 0.73 weeks, respectively. The majority of NT thickness measurements fell within the range of 3 to 4.9 mm, comprising 80.5% of the cases. Furthermore, maternal age was predominantly concentrated in the 20- to 34.9-year age group, accounting for 86.5% of the sample, while pregnant women of advanced maternal age (≥35 years) constituted 10.4% of the participants.

**Table 1:** Incidence of increased NT thickness.

Year	Fetuses	NT ≥ 3.0 mm	Percentage
2017	16,0231	451	0.28%
2018	23,0920	1,010	0.44%
2019	22,4,017	1,141	0.51%
2020	20,4,349	1,220	0.60%
2021	18,9,588	1,077	0.57%
Total	100,9,109	4,899	0.49%

**Table 2:** Characteristics of the 4,200 pregnancies screened with increased nuchal translucency (NT).

Parameter	No. (%) or mean (SD)
Crown-rump length (CRL)	59.15 (8.56)
Gestational age (weeks)	12.33 (0.73)
NT value distribution(mm)	
3.0–4.9	3,380 (80.5)
5.0–6.9	546 (13.0)
7.0–8.9	228 (5.4)
≥9.0	46 (1.1)
Maternal age distribution (years)	
<20	130 (3.1)
20.0–24.9	931 (22.2)
25.0–29.9	1,598 (38.0)
30.0–34.9	1,106 (26.3)
≥35.0	435 (10.4)

### Pregnancy outcomes

Pregnancy outcomes can be classified into two groups: 2,875 patients experienced normal pregnancies without defects (68.5%), while 1,305 patients had adverse outcomes, which included elective termination of pregnancy (TOP), spontaneous abortion (SA), therapeutic abortion (TA), and live births with malformations (31.5%; Table 3). The nuchal translucency (NT) thickness of fetuses in the adverse outcome group (5.05 ± 1.74) was significantly greater than that of fetuses in the normal group (3.68 ± 0.88, *p* < 0.0001). Furthermore, the average maternal age in the adverse outcome group (29.13 ± 5.73) was also higher than that in the normal group (27.60 ± 4.74, *p* < 0.0001). In addition, receiver operating characteristic (ROC) curve analysis demonstrated that NT thickness was a more effective predictor of adverse outcomes (area under the curve [AUC] = 0.760; 95% confidence interval [CI]: 0.747 to 0.773; sensitivity: 0.61; specificity: 0.80) compared to maternal age (AUC = 0.572; 95% CI: 0.557 to 0.587; sensitivity: 0.29; specificity: 0.82; Supplementary Figures S1A–C). The optimal cutoff value for NT thickness associated with an increased incidence of adverse outcomes was determined to be 4 mm. These findings suggest that NT > 4 mm may elevate the risk of adverse pregnancy outcomes.

**Table 3:** Pregnancy outcome of 4,200 fetuses with NT ≥ 3.0 mm.

Follow-up results	Cases	NT thickness	Maternal age
Livebirth with no defects	2875 (68.5)	3.68 ± 0.88	27.60 ± 4.74
TOP/SA/TA/Livebirth with malformation*	1325 (31.5)	5.05 ± 1.74	29.13 ± 5.73

TOP, termination of pregnancy (*n* = 1,027); SA = spontaneous abortion (*n* = 185); TA, threatened abortion (*n* = 93). Livebirth with malformation* include heart defects (*n* = 5); mental retardation and developmental delay (*n* = 3); premature death (*n* = 2); pathogenic gene mutation (*n* = 2); finger/toe defect (*n* = 2); hearing loss (*n* = 1); down syndrome (*n* = 1); congenital leukemia (*n* = 1); congenital esophageal atresia (*n* = 1); diaphragmatocele (*n* = 1); nasal bone absence (*n* = 1). Data are given as Mean ± SD or n (%).

### Ultrasound soft markers

Increased nuchal translucency (NT) thickness is frequently linked to fetal developmental abnormalities. To investigate this association, we summarized the NT ultrasound findings from both the normal and adverse outcome groups (see Table 4). Within the adverse outcome group, we identified 180 patients (13.6%) with significant structural abnormalities, including anencephaly, hydrocephalus, encephalocele, cardiac structural defects, spina bifida, thoracoabdominal defects, visceral eversion, limb developmental abnormalities, and other multisystem disorders. Notably, cardiac structural abnormalities represented the largest proportion, accounting for 3.5%. Furthermore, among the remaining 575 fetuses, we detected abnormal ultrasound soft markers, such as cystic hygroma, anasarca, facial abnormalities, ductus venosus flow abnormalities, and cystic hygroma again, which collectively constituted 43.4%. In contrast, within the normal group comprising 2,875 patients, only 68 (2.4%) exhibited abnormal soft markers. Data analysis suggests that these abnormal ultrasound soft markers may contribute to adverse pregnancy outcomes for fetuses with increased NT (refer to Supplementary Table S2).

**Table 4:** Sonographic findings in fetuses with adverse pregnancy outcomes.

NT diagnosis	No. (%)
**Structural defects**	180 (13.6)
Brain/Head	36 (2.7)
Heart	46 (3.5)
Spine	8 (0.6)
Abdomen	43 (3.2)
Bone/Limb	12 (0.9)
Multiple malformations	35 (2.6)
**Soft indicators**	575 (43.4)
Anasarca	58 (4.4)
Facial abnormality	56 (4.2)
Ductus venosus flow abnormality	85 (6.4)
Hydrothorax	15 (1.1)
Diaphragmatic hernia	5 (0.4)
Cystic hygroma	267 (20.2)

### Karyotype analysis

Pregnant women with increased nuchal translucency (NT) thickness are recommended to undergo amniocentesis for prenatal diagnosis through karyotype analysis. We collected and analyzed the karyotype results of 547 fetuses that underwent this diagnostic procedure. Among these fetuses, 450 (82.3%) exhibited normal karyotypes, while 97 (17.7%) displayed abnormal karyotypes, as detailed in Table 5. The identified abnormalities primarily consisted of aneuploidies, including trisomy 21 (T21), trisomy 18 (T18), trisomy 13 (T13), trisomy 9 (T9), and sex chromosome abnormalities. Notably, T21, commonly known as Down syndrome, represented the largest proportion of abnormalities, accounting for 7.1%. Within the cohort of 450 patients with normal karyotypes, 433 had normal pregnancies, whereas 17 chose elective termination of pregnancy (TOP). In contrast, among the 97 patients with abnormal karyotypes, 93 underwent TOP, leaving 4 who had normal pregnancies attributed to benign chromosomal polymorphisms.

**Table 5:** Karyotype diagnosis in 547 fetuses.

NT diagnosis	No. (%)
Normal	450 (82.3)
T21	39 (7.1)
T18	10 (1.8)
T13	1 (0.2)
T9	1 (0.2)
Sex chromosome	5 (0.9)
Others	41 (7.5)

## Discussion

### A comparative analysis of various prenatal screening technologies

The measurement of nuchal translucency (NT), along with the assessment of fetal trunk and head volume, is predominantly conducted during the first trimester. This approach is particularly effective at identifying conditions such as anencephaly, spina bifida, and other significant structural malformations. While the importance of NT screening is well recognized, its indications for detecting chromosomal abnormalities are limited, primarily serving as a preliminary basis for further prenatal diagnostic procedures (21). In contrast, serological screening is typically performed during both the first and second trimesters of pregnancy. Its affordability is a key advantage; however, it primarily screens for trisomies 21, 18, and 13, as well as neural tube defects, and is associated with elevated rates of false positives and false negatives (22). Non-invasive prenatal testing (NIPT), which uses high-throughput sequencing, has gained widespread adoption for the detection of fetal chromosomal aneuploidy. Although its cost is somewhat higher than that of the first two methods, NIPT offers the highest screening accuracy for aneuploidies, such as trisomy 21, achieving approximately 99% accuracy. Nevertheless, challenges such as sequencing read length, coverage limitations, reference bias, and population polymorphism can affect detection performance and early detection capabilities. With advancements in high-resolution technologies and the integration of artificial intelligence, including machine learning, NIPT is expected to provide more comprehensive genetic data, thereby facilitating the screening of copy number variations (CNVs) and single-gene variations that may be pathogenic or potentially pathogenic to the fetus (23–25). Importantly, the combination of these three screening methods can significantly enhance the accuracy of chromosomal abnormality detection (26–28).

### NT thickness, maternal age, and fetal pregnancy outcomes

Increased nuchal translucency (NT) is positively correlated with the risk of fetal developmental abnormalities; hence, NT ultrasound examination is widely used in first-trimester prenatal screening (29). Currently, two primary criteria exist for defining increased NT. In certain countries, increased NT is defined as values exceeding the 95th (30–32) or 99th (33, 34) percentiles of the NT distribution for a crown-rump length (CRL) ranging from 45 to 84 mm. Other countries utilize thresholds of ≥2.5 mm (35, 36), ≥3 mm (37, 38), or 3.5 mm (39, 40) as indicators of increased NT, with NT ≥3 mm being commonly adopted in China (41). Since 2017, free NT screening has been incorporated into government-funded programs, supported by an established electronic medical records system in Henan, China. A study utilizing this system analyzed the incidence of increased NT and its correlation with pregnancy outcomes in over 1 million singleton pregnancies, representing approximately 90% of all pregnant women in the southeastern region of Henan Province. In our study, we identified 4,899 patients with NT ≥3 mm, resulting in an incidence rate of 0.49%, which aligns with previous reports from Shanghai, China (42). Prior studies have demonstrated that increased NT is associated with adverse pregnancy outcomes, including a heightened risk of structural and chromosomal abnormalities. Consequently, a comprehensive follow-up study was conducted on fetuses with increased NT, monitoring their health status from 6 months to 5 years post-birth. Among the 4,200 fetuses with follow-up results, outcomes were categorized into two groups: 2,875 cases of normal pregnancies without defects and 1,305 cases of adverse pregnancy outcomes. The adverse outcomes included elective termination of pregnancy (TOP), spontaneous abortion (SA), threatened abortion (TA), and live births with malformations. In addition, among the 547 fetuses undergoing karyotype prenatal diagnosis, 450 exhibited normal results, while 97 were identified with chromosomal abnormalities. Trisomy 21 emerged as the most prevalent chromosomal abnormality. Follow-up results for the 450 fetuses with normal karyotypes indicated that approximately 96% of these pregnancies proceeded without complications, with only approximately 4% resulting in adverse outcomes. In contrast, nearly all fetuses with chromosomal abnormalities experienced negative pregnancy outcomes. These findings suggest that while increased nuchal translucency (NT) is closely associated with fetal chromosomal abnormalities, using an NT threshold of ≥3 mm for screening still captures a significant number of fetuses with normal chromosomal profiles.

In this study, we used receiver operating characteristic (ROC) curves to evaluate the diagnostic significance of nuchal translucency (NT) thickness and maternal age in predicting adverse fetal outcomes. Our analysis identified optimal threshold values, revealing that an NT thickness greater than 4 mm and a maternal age exceeding 35 years correspond to an approximate 38 and 10% increase in the risk of adverse outcomes, respectively. Furthermore, when both criteria were simultaneously met, the risk of adverse pregnancy outcomes escalated by more than 50%. These findings underscore the potential implications of NT thickness and maternal age in assessing the risk of adverse fetal outcomes.

### Abnormal ultrasound soft markers and fetal pregnancy outcomes

Among 4,200 fetuses with increased nuchal translucency (NT), 180 cases of major developmental abnormalities involving multiple organ systems were identified. The predominant abnormality involved cardiac structures, accounting for 3.5% of cases, which included conditions such as Tetralogy of Fallot, single atrium/ventricle, cardiac ectopia, and various valve defects. Furthermore, abnormal ultrasound soft markers were observed in 43.4% of fetuses within the adverse outcome group, in contrast to only 2.4% in the normal outcome group. These soft markers included anasarca, facial abnormalities, ductus venosus flow abnormalities, hydrothorax, diaphragmatic hernia, and cystic hygroma. Subsequent analyses indicated that the presence of these soft marker abnormalities significantly elevated the risk of adverse pregnancy outcomes in fetuses with increased NT, as corroborated by the existing literature. For instance, the absence of the nasal bone has been associated with an increased risk of trisomy 21 (43). In addition, abnormal ductus venosus flow is linked to chromosomal abnormalities and congenital heart disease (44, 45). These findings underscore that the coexistence of ultrasound soft marker abnormalities and increased NT substantially heightens the risk of adverse pregnancy outcomes.

## Limitations

This study acknowledges several limitations. First, out of 4,200 fetuses with increased nuchal translucency (NT), only 547 underwent further diagnosis through amniocentesis, revealing that 17.7% exhibited chromosomal abnormalities, with trisomy 21 being the most prevalent at 7.1%. However, these figures may not accurately reflect the overall incidence of chromosomal abnormalities in all fetuses presenting with increased NT. Second, since NT screening is voluntary, women cannot be mandated to pursue additional prenatal testing. Consequently, our analysis was limited to tracking final pregnancy outcomes and assessing risk factors based solely on first-trimester NT screening results. This methodology does not encompass all potential reasons a pregnant woman might choose to terminate a pregnancy. In addition to significant developmental issues and chromosomal anomalies, factors such as psychological stress associated with increased NT, abnormal ultrasound soft markers, the risks linked to amniocentesis, financial implications, maternal comorbidities, lifestyle factors, and familial or social considerations may all play crucial roles in the decision-making process regarding elective termination of pregnancy (TOP) in the context of increased NT (46, 47). Therefore, enhancing public awareness, providing psychological support, and fostering family care for pregnant women could potentially improve outcomes for pregnancies characterized by increased NT.

## Conclusion

Nuchal translucency (NT) ultrasound screening is a valuable tool for pregnant women, as it enhances their understanding of early fetal development. This screening provides critical insights that are essential for maintaining healthy pregnancies and for conducting prenatal diagnoses to rule out malformations. Our study demonstrated a significant correlation between increased NT measurements and a heightened risk of adverse pregnancy outcomes, encompassing both structural and chromosomal abnormalities. In addition, NT values exceeding 4 mm, along with the presence of abnormal ultrasound soft markers emerged as the primary risk factors associated with adverse pregnancy outcomes. These factors should be regarded as essential indicators for prenatal diagnosis in cases of elevated NT.

## Data Availability

The raw data supporting the conclusions of this article will be made available by the authors, without undue reservation.
