# Corrigendum: Pregnancy outcomes of 4,200 fetuses with increased nuchal translucency in Henan, China

**DOI:** 10.3389/fmed.2025.1606281

**Published:** 2025-04-29

**Authors:** Zhenglong Guo, Wenke Yang, Qiongrui Zhao, Yuwei Zhang, Xin Wang, Jinming Wang, Ruili Wang, Bingtao Hao, Shixiu Liao

**Affiliations:** ^1^Henan Provincial Key Laboratory of Genetic Diseases and Functional Genomics & National Health Commission Key Laboratory of Birth Defects Prevention, Medical Genetics Institute of Henan Province, Henan Provincial People's Hospital, People's Hospital of Zhengzhou University, Zhengzhou, China; ^2^Department of Ultrasound, Henan Provincial People's Hospital, Zhengzhou, China

**Keywords:** nuchal translucency thickness, pregnancy outcome, first-trimester screening, chromosome abnormalities, ultrasound soft indicators

In the published article, there was an error in Table 3 as published. There is an error in the statistical values reported in “Cases, NT thickness, and Maternal age”. The corrected Table 3 and its caption appear below.

**Table 3:** Pregnancy outcome of 4,200 fetuses with NT ≥ 3.0 mm.

**Follow-up results**	**Cases**	**NT thickness**	**Maternal age**
Livebirth with no defects	2875 (68.5)	3.68 ± 0.88	27.60 ± 4.74
TOP/SA/TA/Livebirth with malformation^*^	1325 (31.5)	5.05 ± 1.74	29.13 ± 5.73

TOP, termination of pregnancy (*n* = 1,027); SA, spontaneous abortion (*n* = 185); TA, threatened abortion (*n* = 93). Livebirth with malformation^*^ include heart defects (*n* = 5); mental retardation and developmental delay (*n* = 3); premature death (*n* = 2); pathogenic gene mutation (*n* = 2); finger/toe defect (*n* = 2); hearing loss (*n* = 1); down syndrome (*n* = 1); congenital leukemia (*n* = 1); congenital esophageal atresia (*n* = 1); diaphragmatocele (*n* = 1); nasal bone absence (*n* = 1). Data are given as Mean ± SD or *n* (%).

The authors apologize for this error and state that this does not change the scientific conclusions of the article in any way. The original article has been updated.

