# Supplementary material for: Pregnancy outcomes of 4,200 fetuses with increased nuchal translucency in Henan, China
Source: Front Med (Lausanne). 2025 Apr 2;12:1514504. doi: 10.3389/fmed.2025.1514504 (PMC12000101; doi:10.3389/fmed.2025.1514504)
Supplement: Supplementary file 1 [file Data_Sheet_1.docx]

| **Table S1.** The impact of maternal age and NT thickness on pregnancy outcomes | | | | |
| --- | --- | --- | --- | --- |
| **Group*** | **Normal** | **Adverse** | **χ^2^** | **P** |
| 0 | 1895 (83.5%) | 374 (16.5%) | 717.91 | <0.001 |
| 1 | 393 (33.5%) | 142 (26.5%) |  |  |
| 2 | 469 (45.6%) | 560 (54.4%) |  |  |
| 3 | 118 (32.2%) | 249 (67.8%) |  |  |
| Data are given as n (%). Group 0 (age<31, NT<4); Group 1 (age>31, NT<4); Group 2 (age<31, NT>4); Group 3 (age>31, NT>4), *There is a significant diference in comparison between any two groups. | | | | |
