# Supplementary material for: Pregnancy outcomes of 4,200 fetuses with increased nuchal translucency in Henan, China
Source: Front Med (Lausanne). 2025 Apr 2;12:1514504. doi: 10.3389/fmed.2025.1514504 (PMC12000101; doi:10.3389/fmed.2025.1514504)
Supplement: Supplementary file 2 [file Data_Sheet_2.docx]

| Table S2. The impact of ultrasound soft indicators on pregnancy outcomes | | | | | |
| --- | --- | --- | --- | --- | --- |
| Indicator* |  | **Normal** | **Adverse** | **χ^2^** | **P** |
| Anasarca | - | 2862 (69.3%) | 1267 (30.7%) | 94.09 | <0.001 |
|  | + | 13 (18.3%) | 58 (81.7%) |  |  |
| Ductus venosus flow abnormality | - | 2870 (69.8%) | 1240 (30.2%) | 168.48 | <0.001 |
|  | + | 5 (5.6%) | 85 (94.4%) |  |  |
| Hydrothorax | - | 2872 (68.7%) | 1310 (31.3%) | 22.45 | <0.001 |
|  | + | 3 (16.7%) | 15 (83.3%) |  |  |
| Facial abnormality | - | 2873 (69.4%) | 1269 (30.6%) | 115.08 | <0.001 |
|  | + | 2 (3.4%) | 56 (96.6%) |  |  |
| Diaphragmatic hernia | - | 2875 (26.5%) | 1320 (31.5%) | _ | 0.003^a^ |
|  | + | 0 (0.0%) | 5 (100.0%) |  |  |
| Cystic hygroma | - | 2830 (67.8%) | 1058 (27.2%) | 445.60 | <0.001 |
|  | + | 45 (14.4%) | 267 (91.8%) |  |  |
