# Supplementary material for: Pregnancy outcomes of 4,200 fetuses with increased nuchal translucency in Henan, China
Source: Front Med (Lausanne). 2025 Apr 2;12:1514504. doi: 10.3389/fmed.2025.1514504 (PMC12000101; doi:10.3389/fmed.2025.1514504)
Supplement: Supplementary file 3 [file Supplementary_file_1.doc]

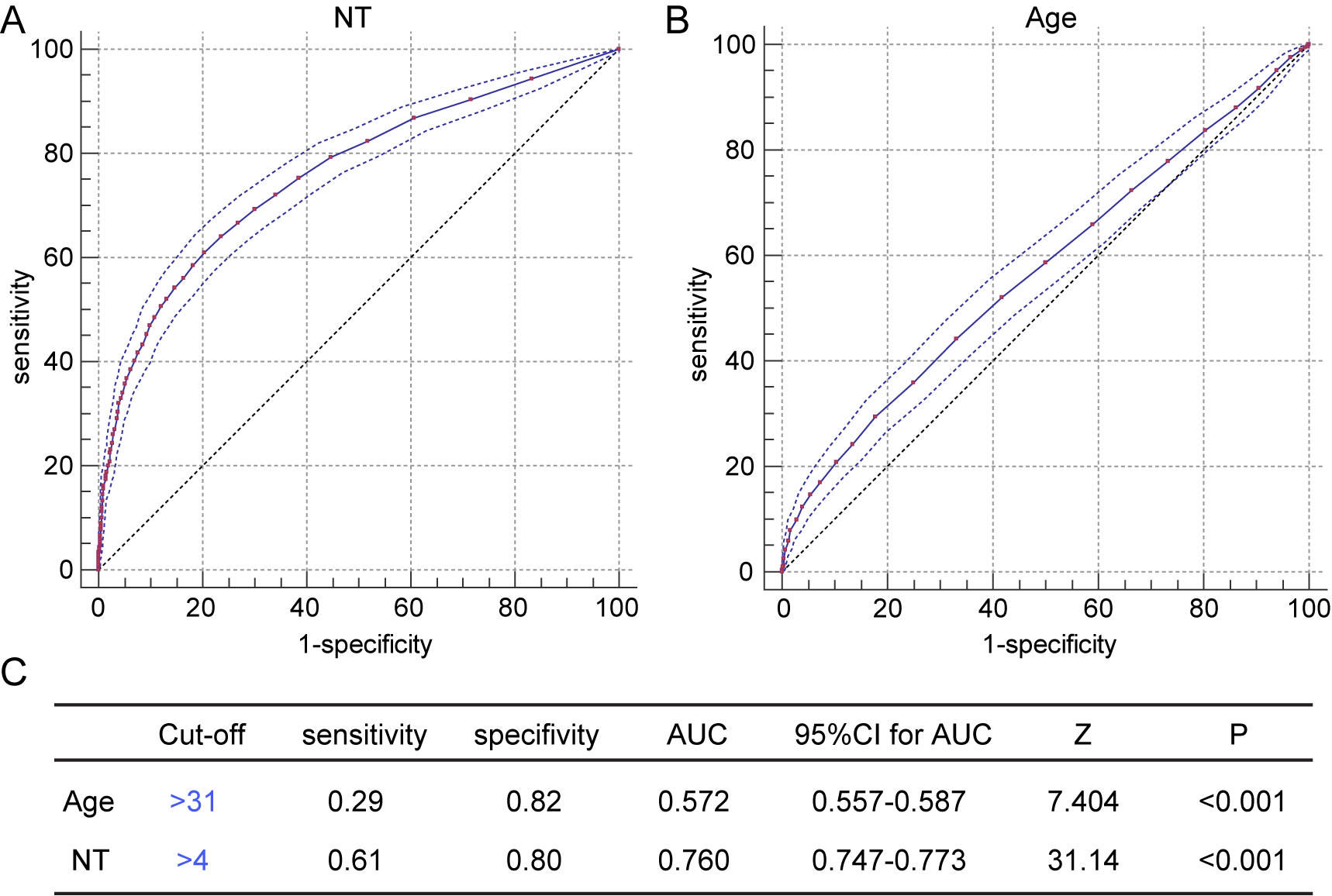


**Figure S1**. **Receiver operating characteristic (ROC) curve analysis for the predictive values of NT thickness and maternal age**. A. Estimated AUC for diagnosis of adverse outcomes with NT thickness. B. Estimated AUC for diagnosis of adverse outcomes with maternal age. C. Diagnosis analysis of adverse outcomes with different indicators.
